# Recombinant human interleukin 4 (IL-4) given as daily subcutaneous injections--a phase I dose toxicity trial.

**DOI:** 10.1038/bjc.1992.243

**Published:** 1992-07

**Authors:** M. H. Gilleece, J. H. Scarffe, A. Ghosh, C. M. Heyworth, E. Bonnem, N. Testa, P. Stern, T. M. Dexter

**Affiliations:** Cancer Research Campaign, Department of Experimental Haematology, Christie Hospital NHS Trust, Manchester, U.K.

## Abstract

Recombinant Interleukin 4 was administered by subcutaneous injection at daily doses of 0.5, 1.0 or 5.0 micrograms kg-1 to nine patients as part of a Phase I Dose Toxicity Study. Dose limiting toxicity was reached at 5 micrograms kg-1 day-1. Symptoms of toxicity included fatigue, 'flu like symptoms and elevated liver enzymes. Modest but significant elevations of neutrophil and platelet counts occurred. No clear evidence of antitumour effects emerged although pain in metastatic lymph nodes and a small fall in myeloma paraprotein levels during dosing were observed. In vitro and murine in vivo studies indicate that patients with lymphoproliferative disease should be selected for Phase II trials.


Br. J. Cancer (1992), 66, 204-210 © Macmillan Press Ltd., 1992

### Recombinant human interleukin 4 (IL-4) given as daily subcutaneous injections – a phase I dose toxicity trial

M.H. Gilleece[1], J.H.. Scarffe[3], A. Ghosh[2], C.M. Heyworth[1], E. Bonnem[4], N. Testa[1], P. Stern[2] & T.M. Dexter[1]

*Cancer Research Campaign Departments of [1]Experimental Haematology, [2]Immunology, [3]Medical Oncology; [4]Paterson Institute for Cancer Research, Christie Hospital NHS Trust, Manchester, Schering-Plough Ltd, New Jersey, USA.*

**Summary** Recombinant Interleukin 4 was administered by subcutaneous injection at daily doses of 0.5, 1.0 or 5.0 μg kg$^{-1}$ to nine patients as part of a Phase I Dose Toxicity Study. Dose limiting toxicity was reached at 5 μg kg$^{-1}$ day$^{-1}$. Symptoms of toxicity included fatigue, 'flu like symptoms and elevated liver enzymes. Modest but significant elevations of neutrophil and platelet counts occurred. No clear evidence of antitumour effects emerged although pain in metastatic lymph nodes and a small fall in myeloma paraprotein levels during dosing were observed. *In vitro* and murine *in vivo* studies indicate that patients with lymphoproliferative disease should be selected for Phase II trials.

The production of growth factors and other cytokines by recombinant DNA technology has facilitated assessment of their *in vivo* therapeutic potential. Alpha Interferon has been used effectively in the treatment of hairy cell leukaemia and maintenance regimens for myeloma, while IL-2 immunotherapy has been used as an alternative to chemotherapy in advanced stages of melanoma or renal cell carcinoma. Granulocyte and Granulocyte-Macrophage Colony Stimulating Factor have recently been shown to be of great promise in ameliorating neutropenia associated with intensive chemotherapy. This paper reports the results of a Phase I dose toxicity clinical trial of Interleukin 4 (IL-4) prior to determining potential anti-neoplastic properties in patients with malignant disease.

IL-4 was first described in 1982 when its role as a distinct B cell growth factor was recognised (Howard, 1982). It is produced mainly by CD4 helper T lymphocytes and exhibits a wide range of effects as an immunoregulatory lymphokine and as a haemopoietic growth factor (Paul, 1991).

During the maturation of early B cells, IL-4 has been shown to induce surface IgM expression and inhibit expression of the primitive B cell marker, CD5 (Hofman *et al.*, 1988; Defrance *et al.*, 1989). IL-4 stimulates B cell proliferation *in vitro* and upregulates CD23, the low affinity IgE receptor, which when secreted can act as a mitogen for B cells (Gordon *et al.*, 1988). It can also upregulate MHC class II antigens (Noelle *et al.*, 1984) and stimulate or inhibit immunoglobulin production and isotype switching to favour IgE production (Snapper *et al.*, 1988).

In addition to its effects on B cell differentiation and development, IL-4 can influence T cell viability and act as a proliferative stimulus to preactivated T cells (Hu Li *et al.*, 1987). IL-4 also stimulates the differentiation of antigen specific cytotoxic T lymphocytes (Horohow *et al.*, 1988) but, in human studies, does not induce lymphokine activated killer (LAK) cell activity. Indeed, IL-4 may stimulate or inhibit Interleukin 2 (IL-2) induced LAK activity although inhibition can in part be overcome by gamma interferon (Han *et al.*, 1988; Spits *et al.*, 1988; Widmer *et al.*, 1987).

IL-4 has pleiotropic effects on various other haemopoietic cells. Combinations of IL-4 with IL-3, G-CSF, erythropoietin, GM-CSF or M-CSF may be stimulatory or inhibitory for myeloid progenitor cells (Jansen *et al.*, 1989; Favre *et al.*, 1990; Broxmeyer *et al.*, 1988).

From these data, it is apparent that IL-4 affects a broad spectrum of cells and it is not surprising that the IL-4 receptor (IL-4R) is expressed by cell lines of several lineages, including lymphoid, myeloid, fibroblast, endothelial and epithelial (Cabrillat *et al.*, 1987; Park *et al.*, 1987).

The receptor is a single high affinity glycoprotein and is maximally upregulated by IL-4 (Zuber *et al.*, 1990).

While the precise physiological functions of IL-4 still remain to be determined, it has emerged that IL-4 has potent anti-tumour effects. These effects have been demonstrated using transplantable malignant cell lines in murine experiments. The tumour cell lines used were characteristically poorly immunogenic and rapidly lethal when injected subcutaneously into mice (Tepper *et al.*, 1989). Transfection of the IL-4 gene into these cells and subsequent expression of IL-4 did not influence their *in vitro* growth characteristics, but prevented *in vivo* growth. The anti-tumour effects exerted by IL-4 were localised to the immediate vicinity of the tumour which was heavily infiltrated with neutrophils, eosinophils and macrophages.

Contralateral non-transfected cells, injected subcutaneously into mice carrying IL-4 transfected tumour cells were not rejected by the mice and showed little or no evidence of infiltration with granulocytes and macrophages. Perilymphatic injection of low doses of IL-4 around lymph nodes draining nontransfected tumours resulted in suppression of tumour development (Bosco *et al.*, 1990) while contralateral simultaneous injection of tumour cells without local IL-4 led to unrestrained proliferation. Mice exposed to tumour and IL-4 were able to reject further challenges with tumour cell alone. Adoptive passive transfer experiments using sublethally irradiated mice suggested that this memory effect was mediated by CD4$^-$ T lymphocytes. The anti-tumour effects of IL-4 do not seem to be confined to experimentally induced tumours since growth inhibition in response to IL-4 has been demonstrated in 56% of freshly isolated specimens of human lymphoid malignancies (Taylor *et al.*, 1990).

The immunomodulatory effects of IL-4 taken in conjunction with the demonstrated anti-tumour effects *in vitro* and *in vivo*, have encouraged the proposal that IL-4 may be of use in the treatment of malignant disease. A Phase I trial was undertaken to evaluate the toxicity and biological effects of IL-4 given at increasing dosage for prolonged periods using the subcutaneous route.

### Materials and methods

### *Study design*

Non glycosylated recombinant human IL-4 (molecular weight 14,000 Da, Schering-Plough Research, Kenilworth, New

Correspondence: J.H. Scarffe, CRC Department of Clinical Oncology, Christie Hospital Trust, Wilmslow Road, Manchester M20 9BX, UK.
Received 14 August 1991; and in revised form 10 February 1992.

Jersey) was supplied in vials containing 75 or 400 μg. Each vial was reconstituted in 1 ml of sterile distilled water. The trial was approved by the local ethical committee. The design of the trial was for each patient to receive a single subcutaneous injection on days 1 (cycle 1) and 8–17 (cycle 2). Those patients who tolerated the drug then went on to receive daily injections on days 29–57 (cycle 3). No IL-4 was given during intervening days. The aim of the trial was for three patients to be entered at each of the following dose levels: 0.5, 1.0, 5.0, 10, 25 $\mu g\ kg^{-1}\ day^{-1}$. There was no dose escalation within the same patient's regimen, and the aim of the study was to establish the dose at which toxicity in any system (WHO grade 3/4) occurred in two out of three patients and was the main limiting factor preventing dose escalation.

*Patients*

Patients over 18 years of age who had given informed consent were entered into the study if they had a malignancy confirmed by histological examination and were unresponsive to conventional therapy. All patients had normal renal function (urea less than 7.5 $mmol\ l^{-1}$, creatinine less than 0.12 $mmol\ l^{-1}$), and adequate bone marrow function (white blood cells more than $3 \times 10^9\ l^{-1}$ and platelets greater than $100 \times 10^9\ l^{-1}$). WHO performance status (Miller *et al.*, 1981) was 0, 1 or 2 and each patient was at least 2 weeks beyond the toxic effects of any prior therapy. Patients were excluded from entry to the trial if they had received any lymphokine within the previous month or any exposure to IL-4. Diabetes mellitus or other condition likely to decompensate under stress, major surgery in the preceding 14 days, acute leukaemia, infection, pregnancy, active gastrointestinal bleeding or intracerebral metastases were further criteria for exclusion.

*Clinical and laboratory monitoring*

Patients attending hospital three times weekly throughout the trial. Regular haematological and biochemical investigations were performed before and during treatment with IL-4. These included differential blood counts, T-cell helper/suppressor ratios, measurement of prothrombin and partial thromboplastin times, biochemistry screen (including liver enzymes, glucose and uric acid), serum immunoglobulins, cholesterol, triglycerides and iron, and urinalysis. Clinical monitoring took the form of physical examination with particular attention to tumour assessment, weight, blood pressure, radial pulse and oral temperature. Periodic electrocardiograms and chest radiograms were performed. Skin testing (multi-test kits, Institut Merieux) was performed pretreatment, and on days 29 and 64 using as challenging antigens, tuberculin, tetanus, glycerin, proteus, candida and streptococcus A. The calculated average diameters of all positive reactions were summated to give a score for each test.

*Clonogenic and IL-4 receptor assays*

Bone marrow aspirates were taken under local anaesthetic from the posterior superior iliac crest for morphological assessment and *in vitro* assays pre-treatment and on days 17, 29 and 57.

Twenty ml of heparinised venous blood were collected on the same days. Bone marrow cells for *in vitro* manipulation were collected in Iscove's medium (Gibco) with 50 units of preservative free heparin (CP Pharmaceuticals Ltd). Three aliquots of this suspension were prepared and clonal assays performed as described previously (Testa, 1985) with minor modifications. Briefly, red cells were removed by sedimentation in 0.1% methylcellulose over 30 min at room temperature. The stromal cell population (CFU-F, Colony Forming unit – fibroblast) was assayed by suspending cells at $1 \times 10^5\ ml^{-1}$ in 5 ml of 15% horse serum (Medical Veterinary Supplies) in Iscove's medium in flasks (Falcon T25) gassed with 5% $CO_2$ and incubating for 10 days at 37°C. The flasks were washed with phosphate buffered saline and the adherent cells fixed in methanol and stained with Crystal Violet. Colonies containing more than 50 fibroblasts were scored. The Ficoll (Flow) separated mononuclear fraction of the second aliquot of bone marrow cells was plated at $10^5$ cells $ml^{-1}$ in 1.2% methylcellulose, 10% conditioned medium from the 5637 bladder carcinoma cell line (as a source of colony stimulating factors, Myers *et al.*, 1984), 2 units of recombinant erythropoietin (Terry Fox Lab), 1% bovine serum albumin (Sigma) and 30% foetal calf serum (Flow Laboratories) in Iscove's medium. Colonies of more than 50 cells were scored as BFU-E (Burst forming unit – erythroid), GM-CFC (Granulocyte macrophage-colony forming cells) and Mix-CFC (Mixed lineage – colony forming cells).

The remaining fraction of bone marrow cells was assayed for IL-4R using a method previously described (Heyworth *et al.*, 1991). Briefly, aliquots (200 μl) containing $3 \times 10^6$ cells and $^{125}$I-labelled IL-4 Specific Activity 1000–1030 Ci $mmol^{-1}$ (gift from Amersham International) at final concentrations of 5–300 pM, were incubated at 26°C for 2 h in the presence or absence of a 30-fold excess of unlabelled IL-4 (gift from Amersham International)

Scatchard analysis was performed to check for the presence of high affinity IL-4R. MLA-144 (Rabin *et al.*, 1981) a cell line known to express high numbers of IL-4R was grown in 10% foetal calf serum in RPMI-1640 (Gibco) and used to confirm the specificity of the $^{125}$I IL-4.

*Immunological assays*

Venous blood taken pre-treatment and on days 15, 29 and 57 was used to assess the immunological responses to IL-4. Peripheral blood mononuclear cells (PBMC) were isolated from 40 ml of heparinised blood by lymphocyte separation medium (Flow Laboratories).

Fresh PBMC were effectors in a 4 h $^{51}$Cr release assay to assess the level of NK and *in vivo* LAK activity against K562, an erythroleukaemia cell line (NK sensitive and LAK sensitive) and Daudi, a Burkitt's lymphoma cell line (NK resistant and LAK sensitive). LAK cells generated *in vitro* were subjected to a similar assay to assess the ability of LAK precursors to become activated. PBMC were assayed in triplicate at effector to target cell ratios of between 40:1 and 10:1 with $5 \times 10^3$ $^{51}$Cr labelled target cells per well. Cytotoxicity was calcualted according to the formula:

$$\%\ \text{cytotoxicity} = \frac{^{51}\text{Cr release test} - \text{spontaneous}\ ^{51}\text{Cr release}}{\text{maximum}\ ^{51}\text{Cr release} - \text{spontaneous}\ ^{51}\text{Cr release}}$$

Spontaneous release was obtained by incubating target cells with medium only and maximum release by incubation of target cells with Triton X-100. A more detailed description of this method is given by Ghosh *et al.* (1989).

LAK cells were generated by incubating $2 \times 10^6\ ml^{-1}$ PBMC with 200 $\mu\ ml^{-1}$ IL-2 (Cetus, Emeryville CA) in six well plates for 4 days. Cells were then harvested and washed twice before assessment of LAK activity.

PBMC were assessed for their ability to proliferate in the presence of mitogens over the trial period. Three concentrations of each mitogen were placed in 96 well plates in triplicate (100 $\mu l\ well^{-1}$) to which 100 μl of PBMC at $2 \times 10^6\ ml^{-1}$ were added. Following an incubation at 37°C for 3 days the cells received 1 μCi of $^3$H-thymidine 4 h prior to harvesting by a cell harvester (Dynatech) onto glass fibre paper. Radioactive uptake was assessed by scintillation counting and a stimulation index (SI) calculated by the formula:

$$\text{SI} = \frac{\text{Mean of test samples}}{\text{Mean of control samples}}$$

Controls consisted of nine wells of cells with no mitogen. The PBMC were assessed against purified Phytohaemaglutinin (PHA, Wellcome Diagnostics) at 1, 0.1 and 0.001 $\mu g\ ml^{-1}$, Pokeweed mitogen (PWM, Sigma) at 1, 0.1 and 0.01 $\mu g\ ml^{-1}$, Formalinised staphylococcus aureus strain cowan 1 (SAC, Sigma) at 0.1, 0.01 and 0.001% v/v, IL-2 at 200, 40 and 10 $\mu\ ml^{-1}$ and Interleukin-4 IL-4 (Immunex Corporation); at 2000, 1000 and 200 $\mu\ ml^{-1}$.

The phenotype of patients lymphocytes was evaluated by FACS on a Coulter Flow Cytometer. Monoclonal antibodies CD3, CD4, CD8 and CD20 were obtained from Coulter electronics and used in a direct fluorescence method. An indirect immunofluorescence method was used to analyse CD23 purchased from Becton-Dickinson, W6/32 (HLA class 1) purchased from Sera-Lab, HLADr purchased from Ortho Diagnostics and CDW32 purchased from The Binding Site.

*Statistical analysis*

Changes in the differential blood count were assessed using Repeated Measures Analysis of Variance using each dose level as groups and days as repeated measure (BMDP Routine 2V).

### Results

Nine patients were entered into the trial, three each at dose levels 0.5, 1, and 5 $\mu g\, kg^{-1}\, day^{-1}$. Eight had solid tumours, most originating in the gastrointestinal tract, and one had multiple myeloma. Three had received previous chemotherapy, one radiotherapy and five had had palliative surgery (Table I). All patients completed cycle I and II of dosing and five patients completed cycle III.

Two patients at the lowest level of dosing withdrew from the trial because of disease progression and two patients at 5 $\mu g\, kg^{-1}$ were withdrawn at day 24 and day 32 because of WHO grade III toxicity. The injection of IL-4 subcutaneously was well tolerated with no pain or reaction at the sites of infection. Two patients learnt to give their own injections. The remaining patients were given their injections by the District Nurse at home. Generalised symptoms of toxicity, such as anorexia, lethargy and 'flu-like symptoms occurred at all dose levels, but subsided within 24 h of the end of each cycle. Dose increments were associated with symptoms of increasing severity and although toxicity at 0.5 $\mu g\, kg^{-1}$ was mild (WHO grade 0–1), one patient reported visual hallucinations during cycle 3 of dosing. These resolved on changing the timing of the injections from morning to evening. Grade 1–2 toxicity occurred at 1 $\mu g\, kg^{-1}$ except when one patient was inadvertently given twice his usual dose and experienced severe arthralgia for 48 h. At 5 $\mu g\, kg^{-1}$ two patients, 8 and 9, were withdrawn because of grade 3 toxicity. Both were pyrexial after dosing and reported severe arthralgia. Patient 8 developed hepatic encephalopathy, but analysis of stored sera revealed him to have been infected with hepatitis C at least 2 years before entry to the trial and residual liver damage may have been a factor although his liver function improved rapidly on stopping IL-4. Patient 9 experienced photophobia, pain in metastatic lymph nodes and vomiting (Table II).

Five patients had liver disease (3, 4, 5, 7 and 8), and two had bony disease (7 and 8) assumed to be secondary to their malignancy. During IL-4 treatment there was elevation of alkaline phosphatase (eight patients), gamma glutamyl transferase (six patients), alanine transaminase (four patients), aspartate transaminase (three patients) and lactate dehydrogenase (two patients), but no significant changes in serum cholesterol or triglyceride levels. Isoenzyme studies in patient 9 confirmed that the elevated alkaline phosphatase was of liver origin. there were no consistent changs in serum immunoglobulins. The rapidly rising myeloma paraprotein in patient 8 with multiple myeloma fell by 15% after 7 days of IL-4 (Table III). However, he was withdrawn from the trial on day 24 because of toxicity, and his paraprotein subsequently increased.

All five patients with pre-existing liver disease showed elevation of prothrombin time by up to 4 s more than control during IL-4 dosing, although the prothrombin time fell to normal in two of these patients when IL-4 was stopped. Two patients without evidence of liver metastases had prolongation of the prothrombin time by 2 s. One of these patients was withdrawn due to disease progression while the prothrombin time fell to normal in the other when IL-4 was stopped. The partial thromboplastin time was prolonged by up to 10 s longer than control in those patients with liver metastases and transiently by 3 s in one patient without liver metastases. Despite these alterations in clotting times, while one patient had a haematemesis related to his malignancy, no other patients had major episodes of bleeding. Faecal occult blood specimens were positive at some stage of the trial for five to eight patients but these results bore no obvious relationship out of dosing and in four patients could have been disease related. Significantly, microscopic haematuria was absent in all patients.

There were modest but statistically significant elevations in neutrophils ($P < 0.00005$) and platelets ($P = 0.0024$) during cycle three of dosing. This rise was most evident at day 36. In patient 7 at 5 $\mu g\, kg^{-1}$ the rise in neutrophils and platelets was substantially higher than in patients at 0.5 or 1.0 $\mu g\, kg^{-1}$ (Figures 1 and 2). There were no significant changes in lymphocyte, eosinophil, basophil or monocyte levels and fluctuations in haemoglobin were unrelated to IL-4 dosing.

Bone marrow aspirates, sampled pretreatment and on days

**Table I** Patients entered on Phase I trial of subcutaneous IL-4

| *Patient no.* | *IL-4 $\mu g\, kg^{-1}$* | *Sex* | *Age* | *Diagnosis* | *Previous treatment for malignancy* |
|---|---|---|---|---|---|
| 1 | 0.5 | F | 51 | Adenocarcinoma pancreas | Gastroenterostomy and choledochojejunostomy |
| 2 | 0.5 | M | 39 | Anaplastic carcinoma oesophagus | Six courses: Vincristine 2 mg; Adriamycin 75 mg; Cyclophosphamide 750 mg |
| 3 | 0.5 | M | 56 | Adenocarcinoma | Nil |
| 4 | 1 | M | 49 | Adenocarcinoma pancreas | Whipples procedure |
| 5 | 1 | M | 55 | Adenocarcinoma caecum | Right hemicolectomy<br>5 Fluorouracil 2.5 mg } weekly for<br>αIFN 3 megaunits × 3/wk } 4 months |
| 6 | 1 | M | 57 | Squamous cell carcinoma bronchus | Radiotherapy right upper lobe |
| 7 | 5 | M | 47 | Squamous cell carcinoma oesophagus | Oesophagectomy |
| 8 | 5 | M | 64 | Myeloma | Melphalan and Prednisolone; cyclophosphamide; dexamethasone; vincristine; adriamycin, αIFN |
| 9 | 5 | F | 53 | Squamous cell carcinoma oesophagus | Oesophagectomy |

**Table II** Symptoms of toxicity following IL-4 injections

| *Patient no.* | *Dose mg kg⁻¹* | *Days on trial* | *Reason for withdrawal* | *Toxicity* | *WHO grade of toxicity* |
|---|---|---|---|---|---|
| 1 | 0.5 | 54 | Disease progression | Papular rash over forearms, days 8–10 | 1 |
| | | | | Shivering | – |
| | | | | Headache, 8 h after injection, resolved overnight | 1 |
| 2 | 0.5 | 85 | — | Shivering } 2 h after dosing, | – |
| | | | | Lethargy } days 11–17, 29–57 | 1 |
| | | | | Sweating } for 1 h | – |
| | | | | Visual hallucinations after morning doses during 3rd phase of dosing, stopped when doses changed to evening admin. | – |
| 3 | 0.5 | 25 | Disease progression | Lethargy during dosing period | 1 |
| 4 | 1 | 85 | – | Back pain } 2 h after dosing, | 2 |
| | | | | Shivering } days 12–19 for 15 min | – |
| | | | | Anorexia during dosing period | – |
| 5 | 1 | 85 | – | Shivering – 2½ h after dosing, days 8–17 29–57, variable duration | – |
| | | | | Day 13: Twice usual dose given in error: headache, back pain and shoulder pain | 3 |
| | | | | Anorexia and lethargy during dosing period | 1 |
| 6 | 1 | 85 | – | Lethargy after first dose | 1 |
| 7 | 5 | 85 | – | Lethargy after first dose | 1 |
| 8 | 5 | 24 | Toxicity | Epistaxis, day 1 | – |
| | | | | Arthralgia day 3, day 12 | 3 |
| | | | | Temperature 37.5, day 10–12 | 1 |
| | | | | Confusion, paranoia and encephalopathy, days 17–20 | – |
| 9 | 5 | 32 | Toxicity | Headache, arthralgia | 3 |
| | | | | Photophobia, shivering | – |
| | | | | Nausea | 2 |
| | | | | Temperature 37.5, Pain in affected lymph nodes, days 8–18, 29–31 | 1 |

17, 29 and 57, showed no evidence of malignant infiltration except in patient 8 who had multiple myeloma. No significant morphological changes were observed during the period of the trial. Bone marrow cells were assessed for the presence of clonogenic haemopoietic and stromal precursors. No consistent trends were observed at any dose levels (colony numbers

**Figure 1** Changes to neutrophil counts in peripheral blood associated with IL-4 dosing. Patient identities indicated in brackets on each graph.

range: 0–98 GM-CFC, 0–61 BFU-E, 0–3 CFU-MIX per $2.5 \times 10^4$ cells and 0–65 CFU-F per $5 \times 10^5$ cells).

Bone marrow mononuclear cells from patients 5, 6, 8 and 9 were assayed for IL-4 receptors pretreatment and on days 17 and 57 but there were no detectable levels prior to or following treatment. In contrast, assays of the MLA-144 cell line used as positive control consistently indicated receptor levels of 290–448 per cell and $K_D$ 45–109 pM.

Changes in immune function induced by IL-4 were assessed indirectly but IL-4 did not enhance delayed hypersensitivity reactions as judged by skin testing with defined allergens since in all patients the skin test score fell or remained at zero. The *in vitro* cytotoxicity of PBMC was assayed against the cultured tumour targets K562 (NK sensitive) and Daudi (NK resistant, LAK sensitive) in all patients. Pretreatment cytotoxicity against K562 varied from 2–39% with five patients showing positive cytotoxicity (above 10%). In patients 4 and 6 cytotoxicity towards K562 increased above pretreatment values on day 28 from 22 to 33% and 11 to 42% respectively, but by day 56, values had decreased to 3 and 6%. In all other patients cytotoxicity towards K562 decreased or remained the same over the treatment period. There were no significant changes in cytotoxicity towards Daudi cells during the treatment period. In all nine patients examined, the PBMC contained LAK cell precursors estimated by induction of cytotoxicity against the NK resistant, LAK sensitive target Daudi by incubation with IL-2 *in vitro* for 4 days. There were no significant quantitative changes observed of *in vitro* LAK cell activity in serial blood samples taken throughout the rIL-4 treatment and values were comparable to normal controls, i.e. 60–80%.

To evaluate whether or not *in vivo* IL-4 administration has any effect on proliferative responses, PBMC obtained at various times pre and post treatment were incubated with different doses of PHA, PWM, SAC, IL-2 and IL-4. Two of the three patients at 0.5 $\mu g\,kg^{-1}$ had 2-fold increased proliferative response to PWM on day 15 but no significant increase in response to PHA. Of the three patients who received 1 $\mu g\,kg^{-1}$ IL-4, one had a 3-fold increase in proliferative response to PHA on days 15 and two had 2–4-fold increased proliferative response to PWM but these decreased at the end of the treatment period. Patients at 5 $\mu g\,kg^{-1}$ had

**Figure 2** Changes to platelet counts in peripheral blood associated with IL-4 dosing. Patient identities indicated in brackets on each graph.

no increased proliferative response to PWM or PHA. Responses to SAC were minimal. Six patients, 2 at 0.5 $\mu$g kg$^{-1}$, 3 at 1.0 $\mu$g kg$^{-1}$, and one patient at 5 $\mu$g kg$^{-1}$ had a slightly increased proliferative response to IL-2 on day 15 (2.5–7-fold) and in four of these patients the increases were sustained at the end of the 3rd treatment cycle. Two patients at 5 $\mu$g kg$^{-1}$ had an increased proliferative response to IL-4 at the end of the treatment cycle.

Cell surface markers were analysed on serial samples from nine patients on IL-4 treatment. There were no significant changes in the percentage of CD3 (pan T) positive cells over the treatment period, although in one patient it decreased from 62% to 53% on day 56. There were slight fluctuations in the percentages of CD4 (T helper) and CD8 (T suppressor cytotoxic) positive cells, but no consistent pattern of change was observed. Slight decreases in the percentage of CD20 (pan B) positive lymphocytes were observed on days 15 in 5 patients. Patient 8 showed the greatest decrease from 19% to 8% on day 15. This patient was withdrawn because of toxicity on day 24 and CD20 positive lymphocytes subsequently increased to 32% on day 56 coinciding with a terminal relapse of myeloma disease. In other patients, percentages of CD20 positive cells were not significantly different at the end of the treatment period. SIgM positive cells increased in patient 8 from 4% to 20% coinciding with myeloma relapse,

**Table III** Changes in serum IgG monoclone and % positive CD23 expressing peripheral blood mononuclear cells in patient 8. IL-4 was given on days 1 and 8–17

| *Day* | *IgG monoclone g l$^{-1}$* | *CD23%* |
|---|---|---|
| −23 | 68 | – |
| 0 | 80 | 14 |
| 8 | 60 | – |
| 15 | 58 | 4 |
| 24 | 64 | 10 |
| 30 | 80 | 25 |

but in no other patients were changes observed. The percentage positive fraction of CD23 antigen bound to PBMC fell in patient 8 from 14 to 4 following the first two cycles of IL-4 dosing (Table III). The patient was withdrawn from the trial on day 24 and the percentage positive fraction then rose from 10 on day 24 to 25 on day 30. There were no increases in percentages of Class II MHC positive lymphocytes over the treatment period.

### Discussion

Nine patients entered this trial to assess the toxicity of subcutaneous injections of IL-4. All patients experienced some toxicity but at 0.5 and 1 $\mu$g kg$^{-1}$ day$^{-1}$ these symptoms were mild and did not interfere with dosing. Two of the three patients treated at 5 $\mu$g kg$^{-1}$ day$^{-1}$ developed WHO grade III toxicity. This suggests that the maximum tolerated dose is between 1 and 5 $\mu$g kg$^{-1}$ day$^{-1}$.

The side effects of lethargy, headache, 'flu-like symptoms and arthralgia are similar to the spectrum of effects seen with administration of a variety of other recombinant cytokines. Patient 5 who had previously had Alpha Interferon described almost identical side effects.

Elevation of liver enzymes and prolongation of prothrombin time were provoked by IL-4 dosing and suggested that IL-4 caused transient hepatic damage, particularly in patients with pre-existing liver disease. These elevations were asymptomatic except in patient 8 who had evidence of previous Hepatitis C infection and in this patient IL-4 may have exacerbated virus induced liver damage. Since Alpha Interferon has been shown to control disease activity in Hepatitis C induced chronic liver disease this is an interesting observation, particularly as IL-4 and Interferon frequently have antagonistic actions *in vitro* (Davis *et al.*, 1989).

In this Phase I trial no clear evidence of tumour response was observed although patient 8 who had myeloma, showed a fall in paraprotein and percentage positive membrane bound CD23 coincident with IL-4 treatment. Both rose on withdrawal of IL-4 treatment, when the patient's disease progressed. CD23 has a dual role as a low affinity IgE receptor and as a marker of B cell proliferation (Conrad, 1990; Cairns & Gordon, 1990). The membrane bound protein undergoes autoproteolysis to form soluble CD23 (sCD23) fragments which are capable of stimulating B cell proliferation. IL-4 upregulates membrane bound CD23 expression *in vitro* and synergises with sCD23 in its mitogenic effects on B cells. The fall in membrane bound CD23 in patient 8 is therefore unexpected and may be a manifestation of the abnormal lymphoproliferation occurring in this patient in whom normal proliferative signals would be likely to be misinterpreted or ignored. Since this fall in CD23 expression was accompanied by a fall in paraprotein it is possible that IL-4 achieved some beneficial effect. Such observations justify inclusion of patients with myeloma or other lymphoproliferative diseases in phase II trials.

IL-4 had no significant effects on myeloid or fibroblast clonogenic progenitor cells in the bone marrow. In view of the available data which show both stimulatory and inhibitory effects of IL-4 on these cells *in vitro* it may have been the case that at the doses used in this trial fine regulation of cell production in the haemopoietic system was able to compensate for this stimulus and still provide a balanced production of mature cells. The moderate rise in peripheral neutrophil and platelet counts seen during the middle of cycle 3 of dosing was not reflected in the progenitor assays at the beginning and end of the cycle and this may be a reflection of homeostatic mechanisms. This is supported by the fact that there was no evidence of increase in IL-4-R levels despite the widespread expression of this receptor. Again this may have been due to the low doses used or to the heterogeneity in terms of lineage and varying maturity of the cell population assayed.

LAK cell precursors were present in all patients as demonstrated by *in vitro* induction of LAK cytotoxicity with IL-2.

However, *in vivo* IL-4 administration did not have any measurable effect on NK activity or induce LAK cell activity. This observation is in agreement with *in vitro* studies (Kawakami *et al.*, 1989) on the effects of IL-4 alone on unstimulated PBMC. *In vitro* and *in vivo* IL-2 primed cells can however show increased cytotoxicity and proliferation when combined with IL-4 *in vitro* (Treismann *et al.*, 1990). It has been shown that IL-4 can augment proliferation of lymphocytes in the presence of mitogens or preactivated cells *in vitro* (Spits *et al.*, 1987). In our study, slight increases were observed in the proliferative response to PWM and PHA on d15 midway through a treatment cycle which may indicate activation of the patients lymphocytes *in vivo*. *In vitro* studies have shown IL-4 to inhibit proliferation of lymphocytes when used simultaneously with IL-2, but at the end of the third treatment cycle no inhibition was observed in the proliferative response to IL-2, and in four patients (2, 4, 5 and 6) it was augmented compared to pretreatment values. These observations on the augmentation of proliferative response suggest that IL-4 has stimulatory effects on subsets of lymphocytes *in vivo* and that combination therapy using IL-2 and IL-4 may be of value.

Preliminary data on the use of IL-4 in other Phase I trials have recently appeared. In one study of ten patients a maximum tolerated intravenous dose of 10 $\mu g\,kg^{-1}$ was reached (Mier *et al.*, 1991). Reported toxicities including nausea, vomiting, fatigue, anorexia, headache, dyspnoea, hyponatraemia and prolonged PT or PTT. In only one patient were hepatic enzymes elevated. Another study (Freimann *et al.*, 1991) of 27 patients achieved an intravenous dose of 160 $\mu g\,m^{-2}\,day^{-1}$ (18 patients) and a subcutaneous dose of 5 $\mu g\,kg^{-1}\,day^{-1}$ (nine patients) without reaching a maximum tolerated dose. Common toxicities were fever, sinus congestion and headache.

Asymptomatic elevations in liver enzymes necessitating dose reduction were predominantly a feature of subcutaneous dosing. Pharmacokinetic data for intravenous or subcutaneous routes of administration are not yet available but may explain the difference in hepatotoxicity.

We have observed 5 $\mu g\,kg^{-1}\,day^{-1}$ to be the limiting dose given subcutaneously. While we have seen no clear tumour responses, the transient fall in myeloma paraprotein during IL-4 dosing noted in patient 8 may suggest some susceptibility of this tumour to IL-4. Certainly this would be in line with reports from Maher *et al.* (1990) or responses to IL-4 by two patients with lymphoproliferative disease and the *in vitro* work of Taylor *et al.* (1990). These reports indicate that patients with lymphoproliferative disorders should be selected for Phase II trials.

We are pleased to acknowledge the assistance given by Dr Pumphries, St Mary's Hospital, Manchester, N. Smith, Sr V. Goode, J. Taylor, L. Watmough, E. Mercer, R. Swindell and J. Cartwright of the Christie Hospital Trust and Cliff Spence of Amersham International.

### References

BOSCO, M., GIOVARELLI, M., FORNI, M. & 4 others (1990). Low doses of IL-4 injected perilymphatically in tumour-bearing mice inhibit the growth of poorly and apparently nonimmunogenic tumours and induce a tumour specific immune memory. *J. Imm.*, **145**, 3136.

BROXMEYER, H.E., LU, L., COOPER, S. & 5 others (1988). Synergistic effects of purified recombinant human and murine B cell growth factor-1/IL-4 on colony formation *in vitro* by haemopoietic progenitor cells. *J. Immunol.*, **141**, 3852.

CABRILLAT, H., GALIZZI, J.P., DJOSSOU, O. & 4 others (1987). High affinity binding of human interleukin 4 to cell lines. *Biochem. Biophys. Res. Commun.*, **149**, 995.

CAIRNS, J.A. & GORDON, J. (1990). Intact, 45 kDa (membrane) form of CD23 in consistently mitogenic for normal and transformed B lymphoblasts. *Eur. J. Immunol.*, **20**, 539.

CONRAD, D.H. (1990). FC E RII/CD23: the low affinity receptor for IgE. *Annu. Rev. Immunol.*, **8**, 623.

DAVIS, G.L., BALART, L.A., SCHIFF, E.R. & 7 others (1989). Treatment of chronic hepatitis C with recombinant interferon alpha. A multicentre randomised, controlled trial. Hepatitis Interventional Therapy Group. *N. Engl. J. Med.*, **321**, 1501.

DEFRANCE, T., VANBERVLIET, B., DURAND, I. & 1 other (1989). Human interleukin-4 down regulates the surface expression of CD5 on normal and leukaemic B cells. *Eur. J. Immunol.*, **19**, 293.

FAVRE, C., SAELAND, S., CAUX, C. & 2 others (1990). Interleukin 4 (IL-4) has basophilic and eosinophilic cell growth promoting activity on cord blood cells. *Blood*, **75**, 67.

FREIMAN, J., ESTROV, Z., HOK, K. & 8 others (1991). Phase I studies of recombinant human interleukin 4 (IL-4). *Proceedings of Asco*, Vol **10**, 725.

GHOSH, A.K., DAZZI, H., THATCHER, N. & 1 other (1989). Lack of correlation between peripheral blood lymphokine activated killer (LAK) cell function and clinical response in patients with advanced malignant melanoma receiving recombinant interleukin 2. *Int. J. Cancer*, **43**, 410.

GORDON, J., MILLSUM, M.J., GUY, G.R. & 1 other (1988). Resting B lymphocytes can be triggered directly through the CDW40 (Bp50) antigen. *J. Immunol.*, **140**, 1425.

HAN, X., HOH, K., BALCH, C.M. & 1 other (1988). Recombinant interleukin 4 (RIL-4) inhibits interleukin-2 induced activation of peripheral blood lymphocytes. *Lymphoh. Res.*, **7**, 227.

HEYWORTH, C.M., HAMPSON, J., DEXTER, T.M. & 6 others (1991). Development of multipotential haemopoietic stem cells to neutrophils is associated with increased expression of receptors for granulocyte macrophage colony stimulating factors: altered biological responses to GM-CSF during development. *Growth Factors* (in press).

HOFMAN, F.M., BROCK, M., TAYLOR, C.R. & 1 other (1988). IL-4 regulates differentiation and proliferation of human precursor B cells. *J. Immunol.*, **141**, 1185.

HOROHOW, D.W., CRIM, J.A., SMITH, P.L. & 1 other (1988). IL-4 (B-cell stimulatory factor 1) regulates multiple aspects of influenza virus specific cell-mediated immunity. *J. Immunol.*, **141**, 4217.

HOWARD, M., FARRAR, J., HILFIKER, M. & 4 others (1982). Identification of a T cell derived B-cell growth factor distinct for interleukin 2. *J. Exp. Med.*, **155**, 914.

HU-LI, J., SHERACH, E.M., MIZUGUCHI, J. & 3 others (1987). B cell stimulatory factor I (interleukin 4) is a potent constimulant for normal resting T lymphocytes. *J. Exp. Med.*, **165**, 157.

JANSEN, J.H., WIENTJENS, G.-J.H.M., FIBBE, W.E. & 2 others (1989). Inhibition of human macrophage colony formation by interleukin 4. *J. Exp. Med.*, **170**, 577.

KAWAKAMI, Y., CUSTEN, M.C., ROSENBERG, S.A. & 1 other (1989). IL-4 regulates IL-2 induction of lymphokine activated killer activity from human lymphocytes. *J. Immunol.*, **142**, 3452.

MAHER, D., BOYD, A., MCKENDRICK, J. & 7 others (1990). Rapid response of B-cell malignancies induced by interleukin 4 (IL-4). *Blood*, **76** 10 (Suppl 600A).

MIER, J.W., VACHINO, G., ROBERT, N.J. & 3 others (1991). Phase I evaluation of thrice daily intravenous bolus interleukin-4 (IL-4). *Proceedings of Asco*, **10**, 704.

MILLER, A.B., HOOGSTRATEN, B., STAQUET, M. & 1 other (1981). Reporting results of Cancer Treatment. *Cancer*, **47**, 207.

NOELLE, R., KRAMMER, P.H., OHARA, J. & 2 others (1984). Increased expression of 1a antigens on resting B cells: a new role for B cell growth factor. *Proc. Natl Acad. Sci. USA*, **81**, 6149.

PARK, L.S., FRIEND, D., SASSENFELD, H.M. & 1 other (1987). Characterisation of the human B cell stimulatory factor 1 receptor. *J. Exp. Med.*, **166**, 476.

PAUL, W.E. (1991). Interleukin 4: a prototypic immunoregulatory lymphokine. *Blood*, **77**, 1859–1870.

RABIN, H., HOPKINS, R.F., RUSCETTI, F.W. & 3 others (1981). *J. Imm.*, **127**, 1852.

SNAPPER, C.M., FINKELMAN, F.D. & PAUL, W.E. (1988). Differential regulation of IgG1 and IgE synthesis by interleukin 4. *J. Exp. Med.*, **167**, 183.

SPITS, H., YSSEL, H., TAKEBE, Y. & 6 others (1987). Recombinant interleukin 4 promotes the growth of human T cells. *J. Immunol.*, **135**, 1142.

SPITS, H., YSSEL, H., PALIARD, X. & 3 others (1988). Interleukin 4 inhibits interleukin 2 mediated induction of human lymphokine activated killer cells, but not the generation of antigen specific cytotoxic T lymhocytes in mixed leucocyte cultures. *J. Imm.*, **141,** 29.

TAYLOR, C.W., GROGAN, T.M. & SALMON, S.E. (1990). Effects of interleukin 4 on the in vitro growth of human lymphoid and plasma cell neoplasm. *Blood*, **75,** 114.

TEPPER, R.I., PATTENGALE, P.K. & LEDEN, P. (1989). Murine interleukin and displays potent antitumour activity *in vivo*. *Cells*, **57,** 503.

TESTA, N.G. (1985). Clonal assays for haemopoietic and lymphoid cells *in vitro*. In *Cell Clones: Manual and Mammalian Cell Techniques*. Potten, C.S. & Henry, J.H. (eds), p. 27. Churchill Livingston: New York.

TREISMAN, J., HIGUDI, C.M., THOMPSON, J.A. & 6 others (1990). Enhancement by interleukin 4 of interleukin 2 or antibody-induced proliferation of lymphocytes from interleukin 2-treated cancer patients. *Cancer Res.*, **50,** 1160.

WIDMER, M.B., ACRES, R.B., SASSENFIELD, H.M. & 1 other (1987). Regulation of cytolytic cell populations from human peripheral blood by B cell stimulatory factor 1 (interleukin 4). *J. Exp. Med.*, **166,** 1447.

ZUBER, C.E., GALIZZI, J.P., VALLE, A. & 3 others (1990). Regulation of IL-4 R expression on normal human B lymhocytes. *Eur. J. Immunol.*, **20,** 551.


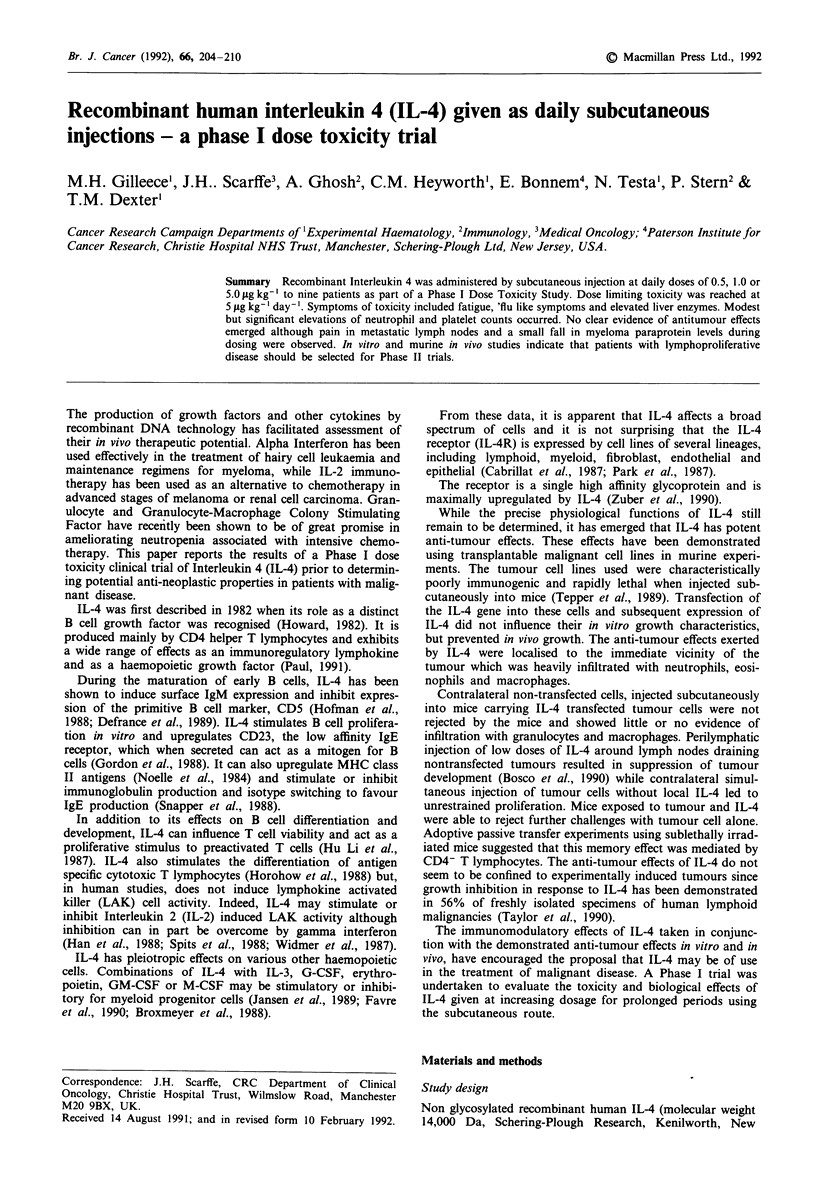

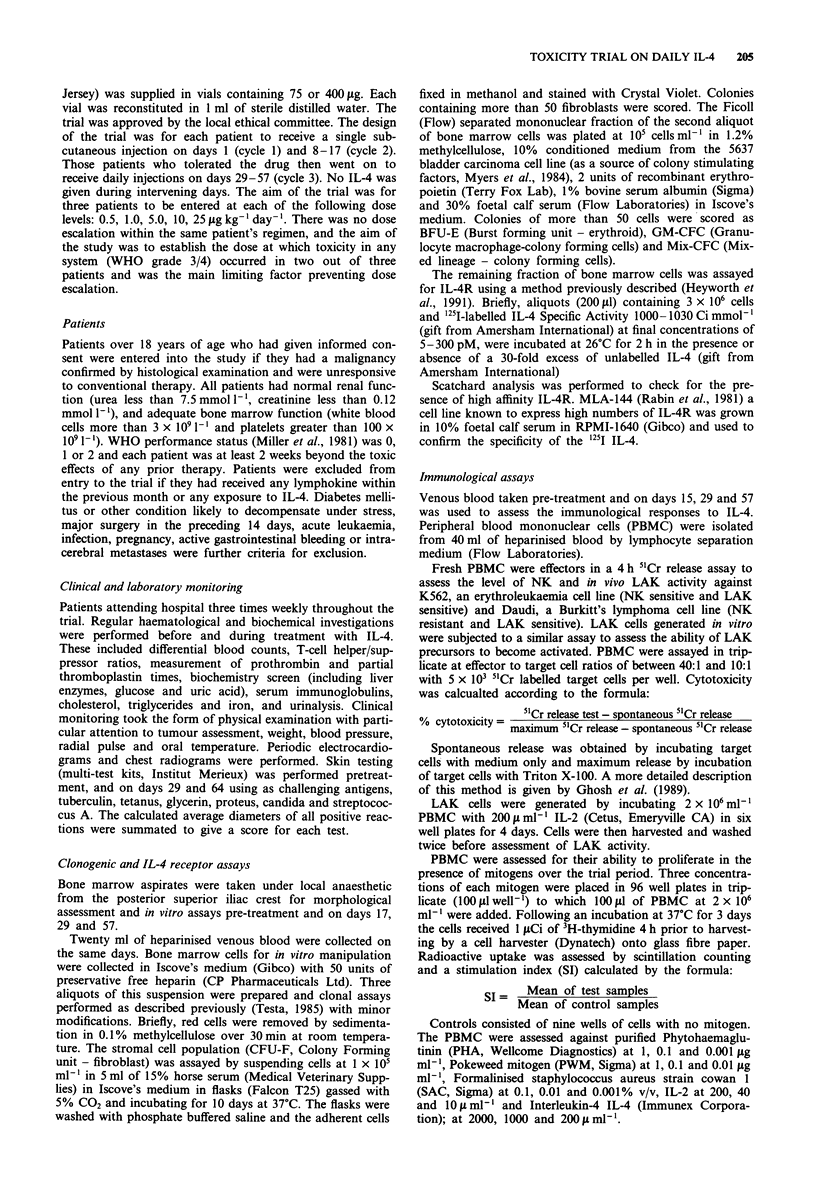

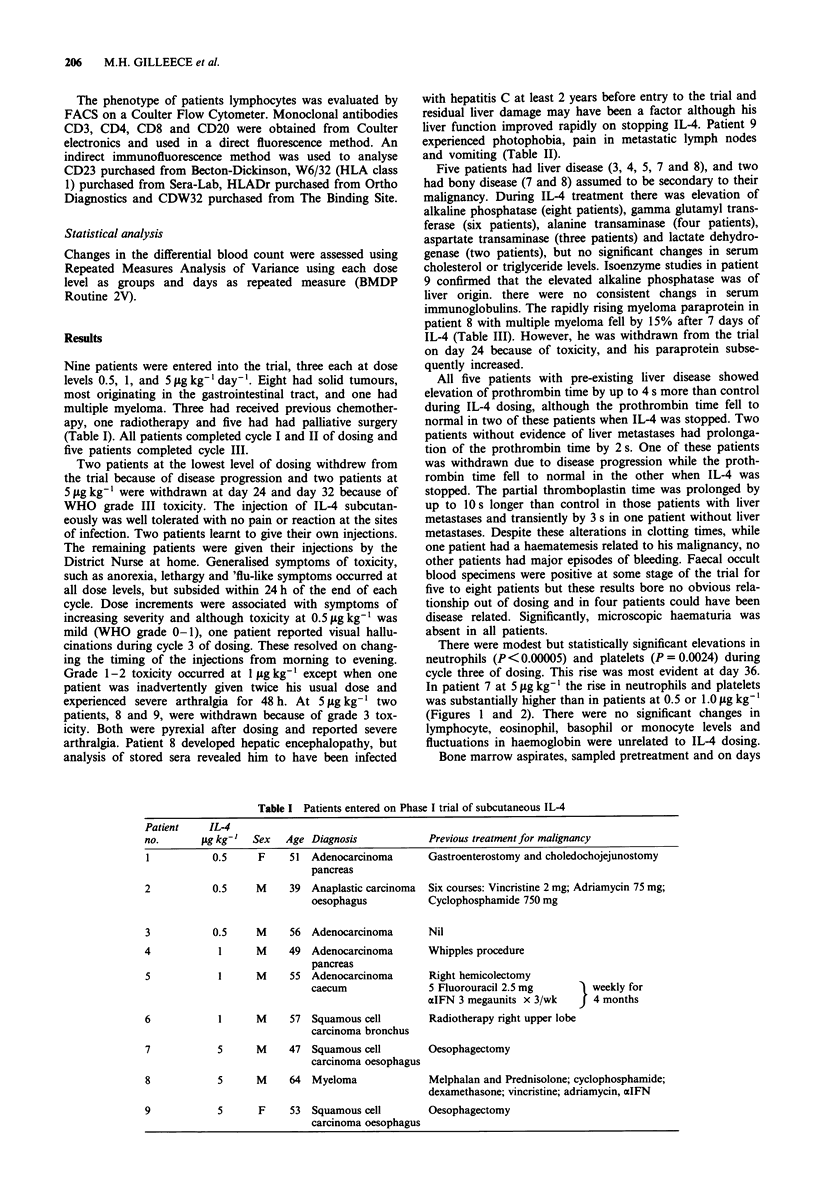

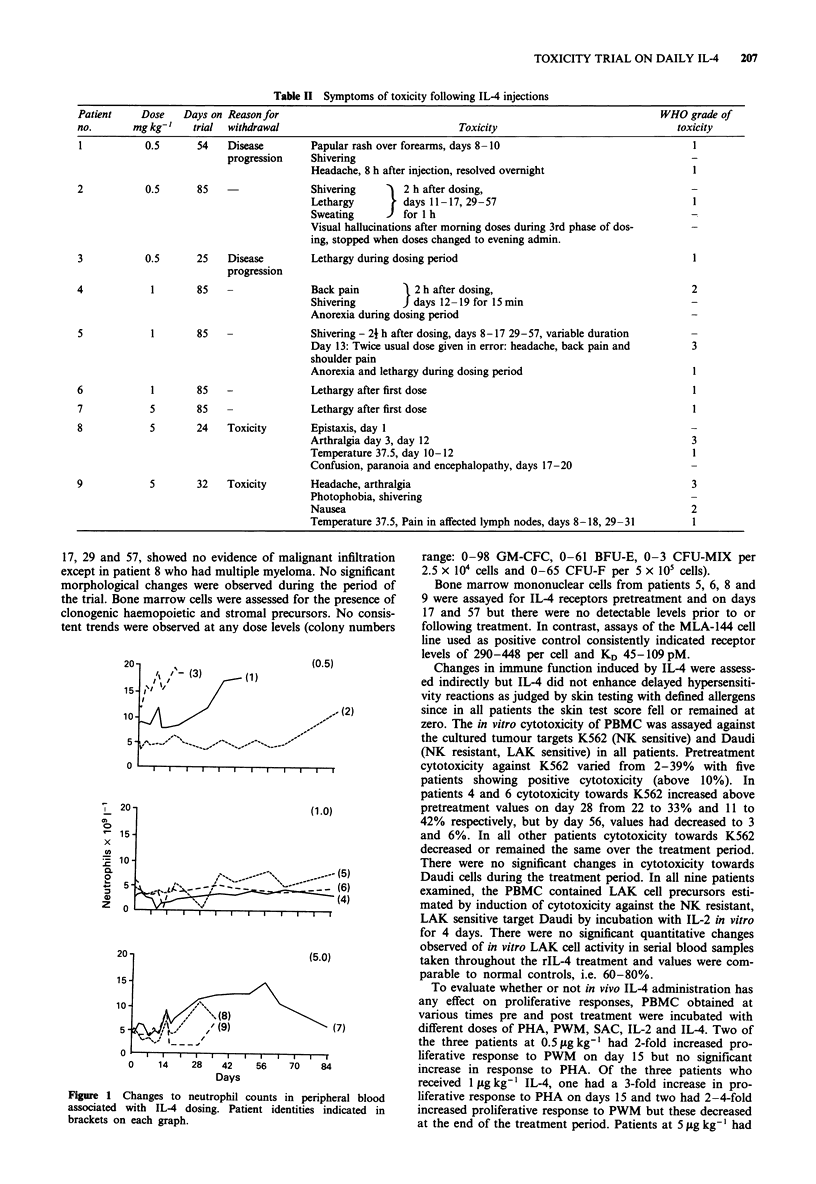

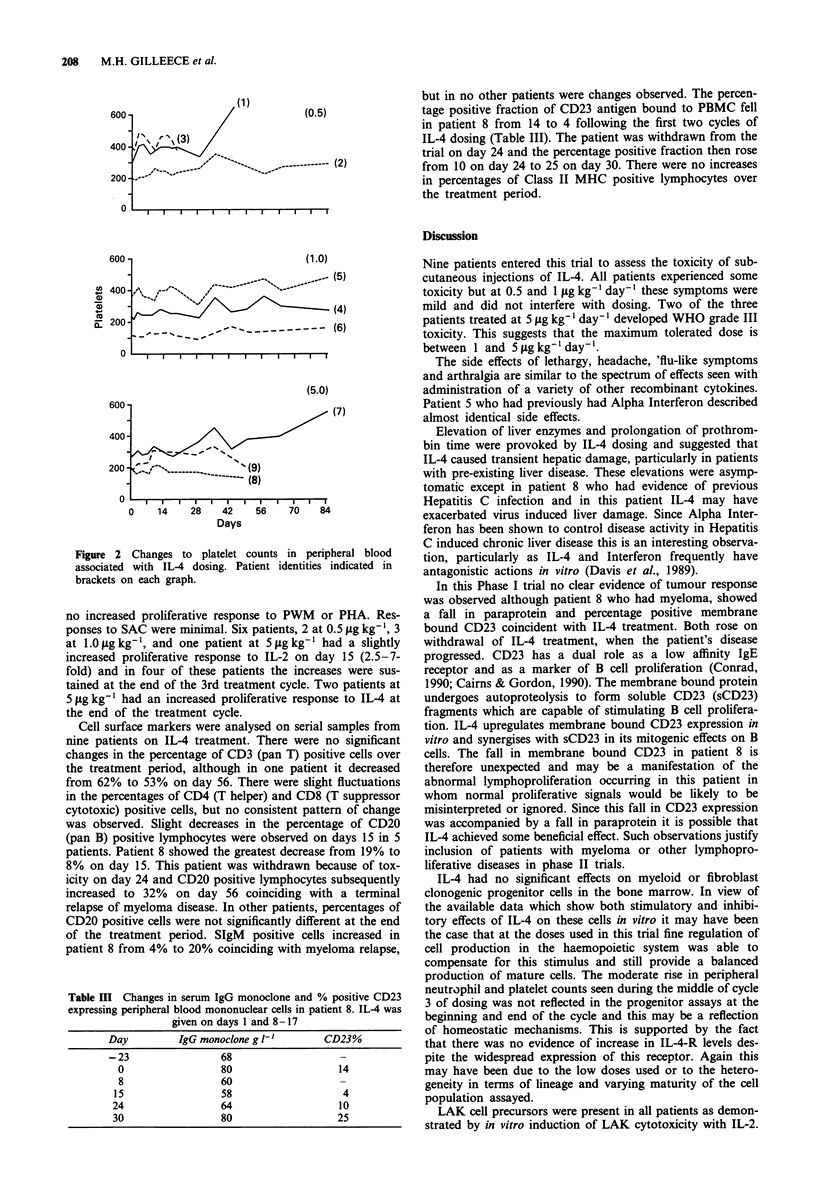

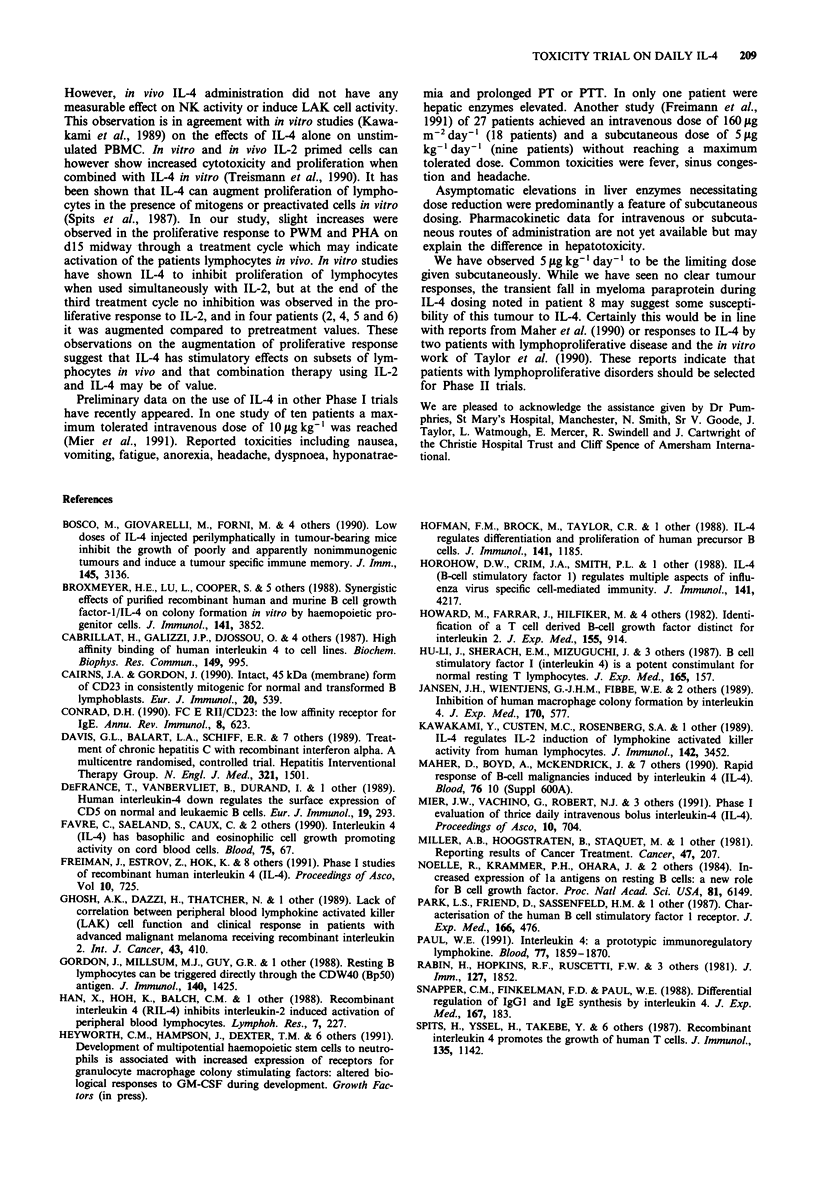

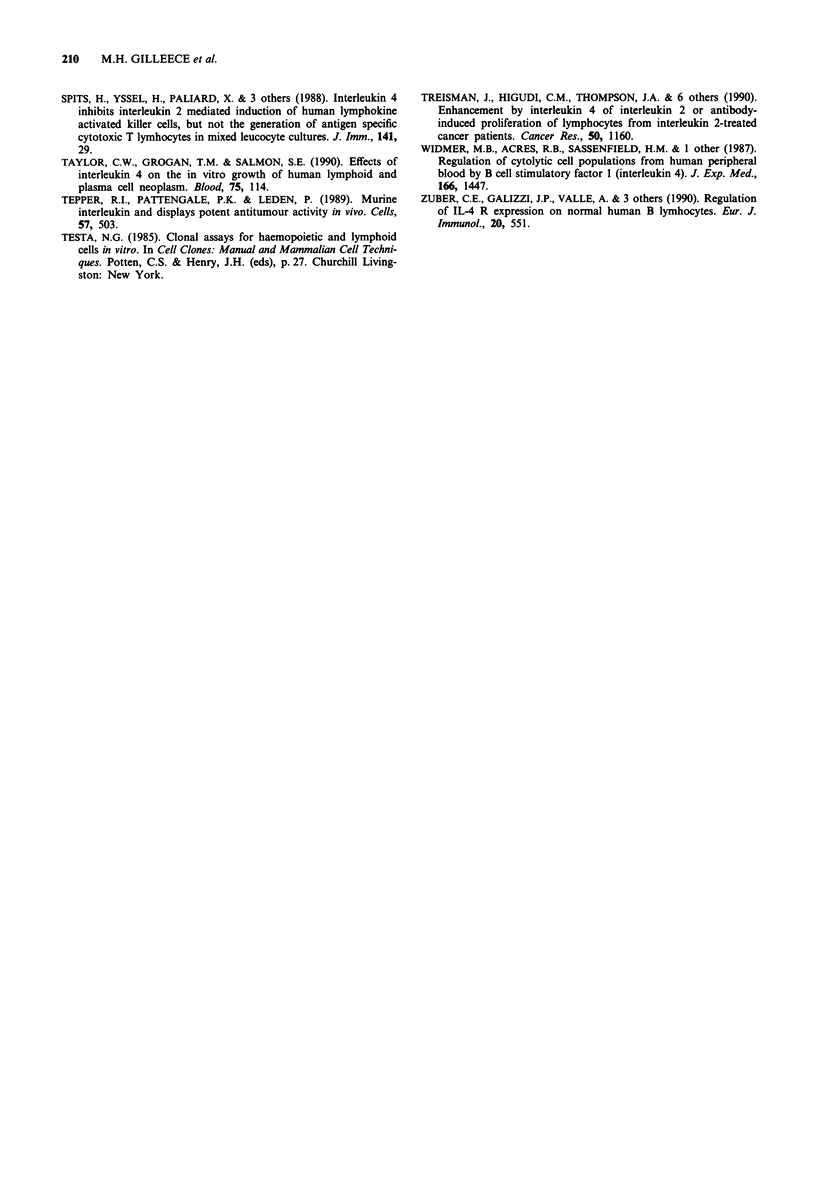


## References

[OCR_00851] Bosco M., Giovarelli M., Forni M., Modesti A., Scarpa S., Masuelli L., Forni G. (1990). Low doses of IL-4 injected perilymphatically in tumor-bearing mice inhibit the growth of poorly and apparently nonimmunogenic tumors and induce a tumor-specific immune memory.. J Immunol.

[OCR_00856] Broxmeyer H. E., Lu L., Cooper S., Tushinski R., Mochizuki D., Rubin B. Y., Gillis S., Williams D. E. (1988). Synergistic effects of purified recombinant human and murine B cell growth factor-1/IL-4 on colony formation in vitro by hematopoietic progenitor cells. Multiple actions.. J Immunol.

[OCR_00862] Cabrillat H., Galizzi J. P., Djossou O., Arai N., Yokota T., Arai K., Banchereau J. (1987). High affinity binding of human interleukin 4 to cell lines.. Biochem Biophys Res Commun.

[OCR_00867] Cairns J. A., Gordon J. (1990). Intact, 45-kDa (membrane) form of CD23 is consistently mitogenic for normal and transformed B lymphoblasts.. Eur J Immunol.

[OCR_00872] Conrad D. H. (1990). Fc epsilon RII/CD23: the low affinity receptor for IgE.. Annu Rev Immunol.

[OCR_00878] Davis G. L., Balart L. A., Schiff E. R., Lindsay K., Bodenheimer H. C., Perrillo R. P., Carey W., Jacobson I. M., Payne J., Dienstag J. L. (1989). Treatment of chronic hepatitis C with recombinant interferon alfa. A multicenter randomized, controlled trial. Hepatitis Interventional Therapy Group.. N Engl J Med.

[OCR_00882] Defrance T., Vanbervliet B., Durand I., Banchereau J. (1989). Human interleukin 4 down-regulates the surface expression of CD5 on normal and leukemic B cells.. Eur J Immunol.

[OCR_00898] Ghosh A. K., Dazzi H., Thatcher N., Moore M. (1989). Lack of correlation between peripheral blood lymphokine-activated killer (LAK) cell function and clinical response in patients with advanced malignant melanoma receiving recombinant interleukin 2.. Int J Cancer.

[OCR_00905] Gordon J., Millsum M. J., Guy G. R., Ledbetter J. A. (1988). Resting B lymphocytes can be triggered directly through the CDw40 (Bp50) antigen. A comparison with IL-4-mediated signaling.. J Immunol.

[OCR_00910] Han X., Itoh K., Balch C. M., Pellis N. R. (1988). Recombinant interleukin 4 (RIL4) inhibits interleukin 2-induced activation of peripheral blood lymphocytes.. Lymphokine Res.

[OCR_00921] Hofman F. M., Brock M., Taylor C. R., Lyons B. (1988). IL-4 regulates differentiation and proliferation of human precursor B cells.. J Immunol.

[OCR_00926] Horohov D. W., Crim J. A., Smith P. L., Siegel J. P. (1988). IL-4 (B cell-stimulatory factor 1) regulates multiple aspects of influenza virus-specific cell-mediated immunity.. J Immunol.

[OCR_00934] Howard M., Farrar J., Hilfiker M., Johnson B., Takatsu K., Hamaoka T., Paul W. E. (1982). Identification of a T cell-derived b cell growth factor distinct from interleukin 2.. J Exp Med.

[OCR_00939] Hu-Li J., Shevach E. M., Mizuguchi J., Ohara J., Mosmann T., Paul W. E. (1987). B cell stimulatory factor 1 (interleukin 4) is a potent costimulant for normal resting T lymphocytes.. J Exp Med.

[OCR_00944] Jansen J. H., Wientjens G. J., Fibbe W. E., Willemze R., Kluin-Nelemans H. C. (1989). Inhibition of human macrophage colony formation by interleukin 4.. J Exp Med.

[OCR_00949] Kawakami Y., Custer M. C., Rosenberg S. A., Lotze M. T. (1989). IL-4 regulates IL-2 induction of lymphokine-activated killer activity from human lymphocytes.. J Immunol.

[OCR_00962] Miller A. B., Hoogstraten B., Staquet M., Winkler A. (1981). Reporting results of cancer treatment.. Cancer.

[OCR_00966] Noelle R., Krammer P. H., Ohara J., Uhr J. W., Vitetta E. S. (1984). Increased expression of Ia antigens on resting B cells: an additional role for B-cell growth factor.. Proc Natl Acad Sci U S A.

[OCR_00970] Park L. S., Friend D., Sassenfeld H. M., Urdal D. L. (1987). Characterization of the human B cell stimulatory factor 1 receptor.. J Exp Med.

[OCR_00975] Paul W. E. (1991). Interleukin-4: a prototypic immunoregulatory lymphokine.. Blood.

[OCR_00979] Rabin H., Hopkins R. F., Ruscetti F. W., Neubauer R. H., Brown R. L., Kawakami T. G. (1981). Spontaneous release of a factor with properties of T cell growth factor from a continuous line of primate tumor T cells.. J Immunol.

[OCR_00983] Snapper C. M., Finkelman F. D., Paul W. E. (1988). Differential regulation of IgG1 and IgE synthesis by interleukin 4.. J Exp Med.

[OCR_00988] Spits H., Yssel H., Takebe Y., Arai N., Yokota T., Lee F., Arai K., Banchereau J., de Vries J. E. (1987). Recombinant interleukin 4 promotes the growth of human T cells.. J Immunol.

[OCR_01007] Tepper R. I., Pattengale P. K., Leder P. (1989). Murine interleukin-4 displays potent anti-tumor activity in vivo.. Cell.

[OCR_01020] Treisman J., Higuchi C. M., Thompson J. A., Gillis S., Lindgren C. G., Kern D. E., Ridell S. R., Greenberg P. D., Fefer A. (1990). Enhancement by interleukin 4 of interleukin 2- or antibody-induced proliferation of lymphocytes from interleukin 2-treated cancer patients.. Cancer Res.

[OCR_01024] Widmer M. B., Acres R. B., Sassenfeld H. M., Grabstein K. H. (1987). Regulation of cytolytic cell populations from human peripheral blood by B cell stimulatory factor 1 (interleukin 4).. J Exp Med.

[OCR_01030] Zuber C. E., Galizzi J. P., Vallé A., Harada N., Howard M., Banchereau J. (1990). Interleukin 4 receptors on normal human B lymphocytes: characterization and regulation.. Eur J Immunol.

